# Inspiration and Insecticide from the Flower Garden

**DOI:** 10.3201/eid2205.AC2205

**Published:** 2016-05

**Authors:** Byron Breedlove, Paul M. Arguin

**Affiliations:** Centers for Disease Control and Prevention, Atlanta, Georgia, USA

**Keywords:** emerging infectious diseases, art and medicine, about the cover, chrysanthemums, Claude Monet, impressionist, inspiration and insecticide from the flower garden, insecticide, malaria, pyrethrum, infectious diseases, vector-borne infections, public health

**Figure:**
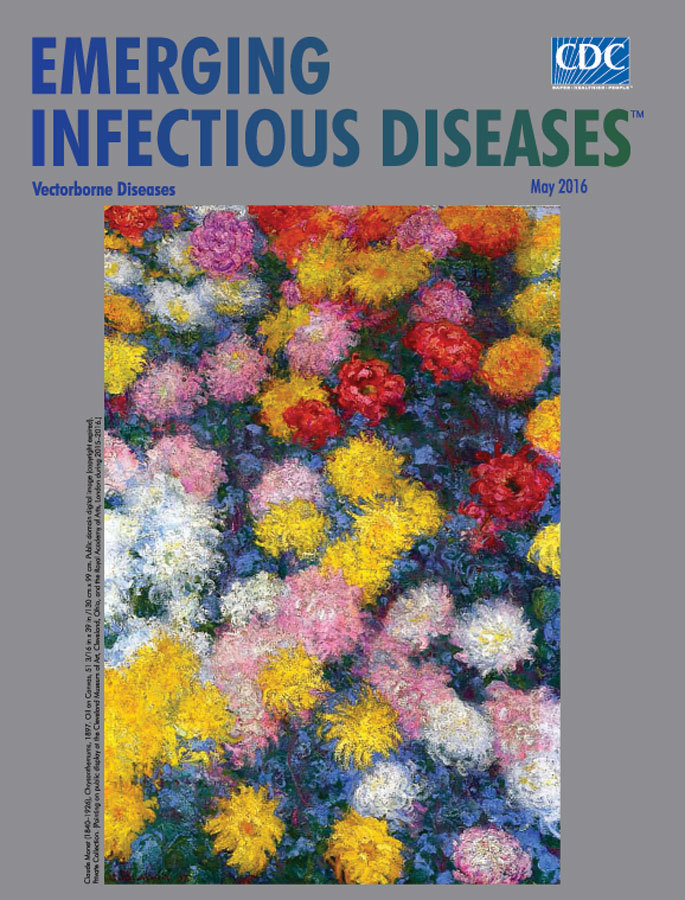
**Claude Monet (1840–1926), *Chrysanthemums*, 1897. Oil on Canvas, 51 3/16 in × 39 in / 130 cm × 99 cm.** Public domain digital image (copyright expired). Private Collection. (Painting on public display at the Cleveland Museum of Art, Cleveland, Ohio, and the Royal Academy of Arts, London during 2015–2016.)

“Like the fires caught and fixed by a great colourist from the impermanence of the atmosphere and the sun, so that they should enter and adorn a human dwelling, they invited me, those chrysanthemums, to put away all my sorrows and to taste with a greedy rapture during that tea-time hour the all-too-fleeting pleasures of November, whose intimate and mysterious splendour they set ablaze all around me.” 

—*In the Shadow of Young Girls in Flower* by Marcel Proust

Perhaps because chrysanthemums bloom well into the autumn and come in an array of colors and varieties, these hardy flowers have inspired poets, writers, and artists for millennia. French Impressionist artist Claude Monet celebrated this flower in a series of four still-life paintings in 1897, including *Chrysanthemum, 1897*, selected for this month’s cover art. These works marked the start of a fresh approach that foreshadowed Monet’s renowned water-lily paintings, breaking from conventional arrangements of foods and flowers characteristic of his still-life paintings from the previous two decades. Art scholar John House wrote that the *Chrysanthemum* canvases are “unconstrained by still-life conventions, covered entirely by a vibrant, virtuoso display of blossoms and foliage.”

In discussing the *Chrysanthemum* series, House noted that “The flowers fill the canvas, with no explicit spatial context. The blooms are arranged in clusters of varied color and texture, placed against more shadowy foliage, which allows their forms to float across the whole picture surface.” Art scholars Robert Gordon and Andrew Forge also commented on Monet’s composition of these paintings, stating that Monet “comes at [the flowers] head on, without a compositional attitude: they are dumped in front of him, bushy or svelte, vivid, teeming with their specific energy, without atmosphere, an explosion.”

“Like the fires caught and fixed by a great colourist,” Monet’s yellow and red flowers heighten this sense of energy, contrasting with the mass of paler white, pink, and lavender flowers. Clustered on stalks with blue-green leaves, the teeming blossoms are viewed from above, as though from an airborne pollinator’s perspective. These chrysanthemums were almost certainly cultivated in the artist’s own legendary gardens at Giverny. During the past 33 years of his life, Monet devoted large amounts of his time and resources to creating and sustaining his gardens, and as one critic remarked of Monet in 1898, “He reads more catalogues and horticultural price lists than articles on aesthetics.”

During Monet’s lifetime, chrysanthemums were relative newcomers to Europe, having not been introduced to the Western world until the 17th century. According to the National Chrysanthemum Society, in 1753 “Swedish botanist Karl Linnaeus, combined the Greek words chrysos, meaning gold with anthemon, meaning flower. . . an accurate description of the ancient species, as it also points out the mum’s need for sunlight.” 

Images that resemble today’s chrysanthemums are found on ancient Chinese pottery from as far back as the 15th century bce. Those images, along with writings from that time, reveal that the Chinese have long cultivated *chu*, the ancient Chinese name for chrysanthemum, as a flowering herb. During the 8th century ace, the chrysanthemum appeared in Japan, where it was called *kiku.* This flower has unusual significance for the Japanese, who adopted a single flowered chrysanthemum—a 16-floret variety called *ichimonjiginu*—as the crest and official seal of their Emperor and celebrate chrysanthemums in an annual Festival of Happiness.

Valued for more than its visual beauty, the chrysanthemum was originally imported to Japan as medicine. According to The National Chrysanthemum Society, its roots were boiled to create a tonic to relieve headaches; its sprouts and petals were added to salads; and its leaves were steeped to make a drink. The insecticidal and insect-repellent properties of some types of chrysanthemums have been recognized for thousands of years. In particular, the Dalmatian chrysanthemum, or *Tanacetum cinerariifolium,* is an important source of the natural botanical insecticide, pyrethrum.

Pyrethrum, a naturally occurring mixture of chemicals found in certain chrysanthemum flowers, kills ticks and insects such as fleas and mosquitos by attacking their nervous systems. Six individual chemical compounds called pyrethrins have active insecticidal and acaracidal properties in the pyrethrum extract.

Pyrethroids are man-made chemicals similar in structure to the pyrethrins but with more useful attributes, including increased toxicity to insects and environmental stability. Those qualities enable their use in products varying from mosquito coils and vaporizers to human and animal medicines that have household, agricultural, and public health applications. Synthetic pyrethroids such as permethrin can be applied to materials to create insect- and tick-repellent clothing and uniforms for preventing infections such as Zika virus disease, leishmaniasis, and African tick bite fever among people, including international travelers, members of the military, or public health workers.

When used as the active ingredient on long-lasting insecticide-treated bed nets hung over the sleeping spaces of persons at risk for vectorborne infections such as malaria, pyrethroids act as a repellent that reduces the numbers of insects that enter the dwelling and as an insecticide that kills insects that come into contact with the net. These mosquito nets, a cornerstone of malaria control programs worldwide, have contributed to recent dramatic reductions in the numbers of cases and deaths.

The life-saving effectiveness of the mosquito net intervention, however, is in danger of being lost, in part, because of the emergence and spread of pyrethroid resistance among mosquitoes. Developing new insecticides and resistance management strategies for their use are essential for continuing public health efforts to achieve and support the 2016 World Malaria Day theme, “End Malaria for Good.”
